# Extracellular Protease ADAMTS1 Is Required at Early Stages of Human Uveal Melanoma Development by Inducing Stemness and Endothelial-Like Features on Tumor Cells

**DOI:** 10.3390/cancers12040801

**Published:** 2020-03-27

**Authors:** Carlos Peris-Torres, María del Carmen Plaza-Calonge, Raúl López-Domínguez, Silvia Domínguez-García, Antonio Barrientos-Durán, Pedro Carmona-Sáez, Juan Carlos Rodríguez-Manzaneque

**Affiliations:** 1 GENYO. Centre for Genomics and Oncological Research: Pfizer/Universidad de Granada/Junta de Andalucía, 114, 18016 Granada, Spain; 2 Department of Statistics and Operational Research, University of Granada, 18071 Granada, Spain

**Keywords:** ADAMTS, cancer stem cell, endothelial-like phenotype, extracellular matrix, vasculogenic mimicry

## Abstract

Extracellular matrix remodeling within the tumor microenvironment has been recognized as a relevant dynamic framework during tumor growth. However, research on proteases that trigger this remodeling keeps revealing a wide range of actions including both pro- and anti-tumorigenic. The extracellular protease *ADAMTS1* exemplifies this dual role. In this work, we first confirmed a positive correlation of *ADAMTS1* with endothelial-like phenotype of human melanoma cells together with the finding of associated signatures, including key genes such as endothelial *CDH5*. Using a CRISPR-Cas9 approach, we observed that the inhibition of *ADAMTS1* in an aggressive uveal melanoma model compromised its endothelial-like properties, and more importantly, caused a robust blockade on the progression of tumor xenografts. Although vasculature emerged affected in *ADAMTS1*-deficient tumors, the most relevant action implied the downregulation of endothelial *CDH5* in tumor cells, in association with stemness markers. Indeed, melanoma sphere assays also revealed a deficient commitment to form spheres in the absence of *ADAMTS1*, directly correlating with stemness markers and, remarkably, also with CDH5. Finally, taking advantage of advanced bioinformatics tools and available public data of uveal melanomas, we disclosed new prognosis factors, including endothelial elements and ADAMTS proteases. Our findings support the key role of ADAMTS proteases for uveal melanoma development since earlier stages, modulating the complex crosstalk between extracellular matrix and the induction of stemness and endothelial-like features. To our knowledge, this is the first report that supports the development of therapeutic targets on the extracellular matrix to overcome uveal melanoma.

## 1. Introduction

The tumor microenvironment (TME) has been highlighted as a key player during tumor development, providing specific signals that support cell invasion, proliferation and phenotypic plasticity [1]. TME is extremely dynamic, involving all its cellular and extracellular constituents, and its full understanding still requires a multilayered research to identify new targets and biomarkers [2]. On the extracellular side, the actions of a variety of proteases contributed to such dynamism by the alteration of multiple pathways with impact during all stages of neoplasias. In fact, proteolytic activity has been widely acknowledged in different tumor types [3]. Among these proteases, studies on ADAMTS1, first member of the ADAMTS (A Disintegrin And Metalloprotease with ThromboSpondin motifs) family [4], underlined its tumor suppressive [5,6] but also its protumorigenic properties [7,8,9], including its key contribution for the acquisition of an endothelial-like (EL) phenotype [10] or its competences to modulate the immune response [11]. Likewise, similar attributes have been reported to further ADAMTS members [12], recognizing the necessary commitment to know better the nature of ADAMTS-mediated actions that concern the modulation of phenotypic properties of cancer cells.

The influence of extracellular matrix (ECM) remodeling on plasticity and stemness capacities of cancer cells is still an open query. For example, the study of vasculogenic mimicry (VM) [13] revealed an alternative mechanism of neovascularization where tumor cells revert to a stem-like state, favoring the acquisition of an EL phenotype [14]. Although few reports have described the contribution of metalloproteases cooperating with ECM factors during this phenomenon [15], ECM remodeling needs to be thoroughly considered, as physical and chemical properties affect cancer plasticity [16]. VM was firstly reported on melanoma cases but many questions remain regarding the relationship between stemness, cancer plasticity and EL phenotype. We now add new insights about the contribution of ECM regulatory molecules in a human uveal melanoma (UVM) setting. Very significantly, although UVM is classified as a rare cancer, it is very aggressive with up to 50% of the patients developing metastasis [17]. Indeed, UVM plasticity has been previously uncovered including the relevant regulation of microenvironment-related pathways [18].

Here, we first demonstrated an in vitro EL phenotype for various melanoma cell lines that correlated with *ADAMTS1* expression, and we also unveiled common gene signatures with endothelial lineages. Moreover, the inhibition of *ADAMTS1* affected in vitro EL attributes and, more importantly, caused a major halt of tumor progression in mice with alterations in vascular and endothelial parameters. We also observed a significant compromise of stemness features in tumor cells, emphasized by our melanoma sphere assays. Finally, taking advantage of advanced bioinformatics tools and available TCGA data on UVM, we disclosed new prognosis elements that sustained our experimental data. To our knowledge, this is the first study reporting the activity of an extracellular protease on the development of UVM by the induction of stemness and endothelial-like features, and it prompts the development of new strategies to fight this fatal malignancy.

## 2. Results

### 2.1. ADAMTS1 Expression Correlates with An Endothelial-Like Phenotype of Melanoma Cells

**Figure 1:**
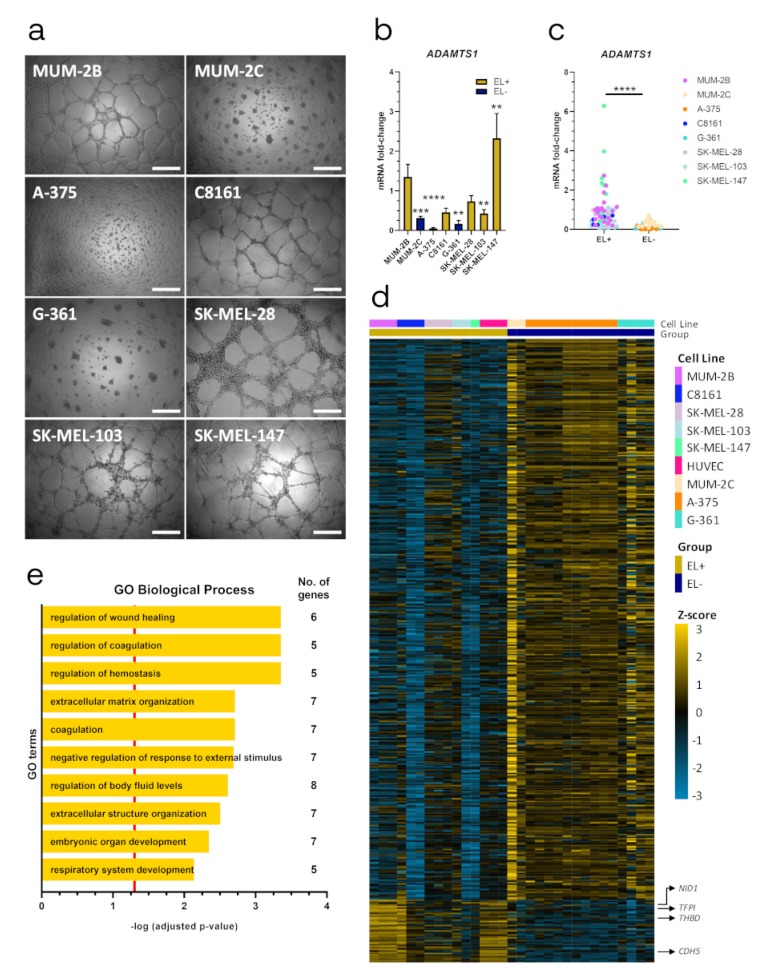
Endothelial-like properties of melanoma cells and correlation with ADAMTS1 expression. (**a**) Representative images of 3D Matrigel-based assay of human melanoma cell lines, 24 h after seeding. 20,000 cells/well were cultured for MUM-2B, SK-MEL-147, C8161, MUM-2C and SK-MEL-103; and 30,000 cells/well for SK-MEL-28, G-361 and A-375 (white scale bar = 500 µm); (**b**) Graph representing mRNA fold change expression of *ADAMTS1* in human melanoma cell lines. Values are relative to MUM-2B (*n* = 21 for MUM-2B, *n* = 17 for MUM-2C, *n* = 11 for A-375, *n* = 5 for C8161 and SK-MEL-28, *n* = 6 for G-361, *n* = 15 for SK-MEL-103 and *n* = 9 for SK-MEL-147). EL+ and EL− phenotypes are indicated; (**c**) Graph representing mRNA fold change expression of *ADAMTS1* in human melanoma cell lines, according to their EL+ or EL− phenotype (values are based in same data that Figure 1C); (**d**) Heatmap showing differential gene expression between EL+ (including HUVECs) and EL− cell lines. Only significant differently expressed genes are depicted (47 upregulated and 420 downregulated, FDR < 0.05). Gene Expression Omnibus (GEO) ID samples are listed and color coded in Table S2; (**e**) Representation of top ten GO Biological Processes after enrichment analysis using significantly upregulated genes in EL+ cells. Red line determined the limit of significance: -log (0.05). (****, *p* < 0.0001; ***, *p* < 0.001; and **, *p* < 0.01).

We used the well-established Matrigel assay [19] to characterize the endothelial-like (EL) phenotype of melanoma cells. While MUM-2B, SK-MEL-28, SK-MEL-103, SK-MEL-147 and C8161 generated clear endothelial-like networks in Matrigel, MUM-2C, A-375 and G-361 formed cell clusters (Figure 1a). Accordingly, we classified these lines as EL+ and EL− cell lines, respectively. We evaluated *ADAMTS1* gene expression in all cell lines (Figure 1b and Figure S1a), observing that its expression was significantly higher in EL+ cell lines compared with EL− ones (Figure 1c).

Taking into account the public availability of gene expression data of these cell lines, we executed an in silico comparison of their gene signatures including also human umbilical vein endothelial cells (HUVECs) to ponder their EL related phenotype (Figure 1d and Table S2). We obtained 467 genes with a significantly different expression between EL+ (including HUVECs) and EL− cells: 47 upregulated and 420 downregulated (Figure 1d and Table S3a). Interestingly, within the upregulated group we found relevant endothelial-related genes (e.g., *CDH5* [VE-cadherin], *TFPI* and *THBD*), which were also linked with ECM remodeling (e.g., *NID1*) in our models. Indeed, GO enrichment analysis revealed a prevalence of pathways strongly related with vascular functionality, such as regulation of coagulation, hemostasis, wound healing and ECM organization (Figure 1e and Table S3b), including some of the genes mentioned above. Overall, these results encourage additional studies of the EL phenotype of our melanoma cells and the contribution of ADAMTS1.

### 2.2. ADAMTS1 Inhibition Affects In Vitro Endothelial-like Phenotypic Properties and Endothelial-Related Signature

**Figure 2:**
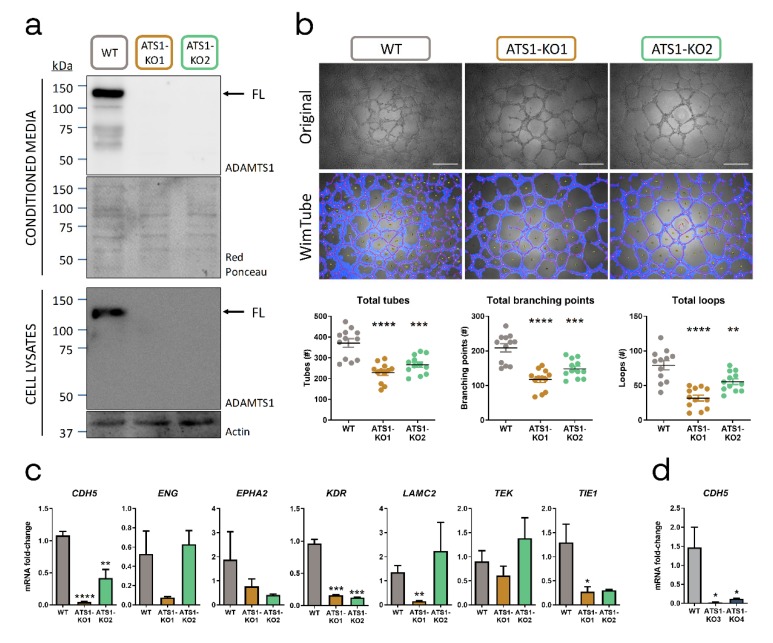
ADAMTS1 inhibition affects in vitro endothelial-like phenotypic properties and endothelial-related signature. (**a**) Western blot analysis of conditioned media and cell lysates of ADAMTS1 in MUM-2B WT and ATS1-KO cells. Black arrows point full-length (FL) ADAMTS1. Red Ponceau staining and Actin were used as loading controls for conditioned media and cell lysates, respectively (uncut blots including a densitometry analysis are shown in Figure S1c); (**b**) Representative images (original and WimTube filtered) of Matrigel assay for MUM-2B WT and ATS1-KO cells, 24 h after seeding 20,000 cells/well. Scatter plots represent the parameters resulting of WimTube analysis: total tubes, total branching points and total loops (*n* = 12 for all groups, white scale bar = 500 µm); (**c**) Graphs representing mRNA fold change expression of *CDH5*, *ENG*, *EPHA2*, *KDR*, *LAMC2*, *TEK* and *TIE1* in MUM-2B WT and ATS1-KO cells (*n* = 3–5 for WT, *n* = 3–6 for ATS1-KO1 and *n* = 2–4 for ATS1-KO2); (**d**) Graph representing mRNA fold change expression of *CDH5* in C8161 WT and ATS1-KO cells (*n* = 4 for all groups). (****, *p* < 0.0001; ***, *p* < 0.001; **, *p* < 0.01; and *, *p* < 0.05. WT cells were used as control for statistical analyses).

We evaluated if the inhibition of ADAMTS1 altered EL+ phenotypic plasticity in uveal MUM-2B cells, using CRISPR-Cas9 technology. Once confirmed ADAMTS1 edition and inhibition in two different MUM-2B clones by Sanger sequencing (Figure S1b) and Western blot (Figure 2a and Figure S1c), we studied their EL phenotype performing in vitro Matrigel assays (Figure 2b). We evaluated the resulting structures with the non-biased WimTube tool. Using different cell culture densities, we confirmed that ADAMTS1 inhibition diminished the number of tubes, branching points and loops (Figure 2b and Figure S1d).

Following these findings, we then wondered if the impairment of the EL phenotype in MUM-2B ADAMTS1-knock out (ATS1-KO) cells was also reflected on their endothelial-related signature. We compared the expression levels of recognized endothelial-related genes that are relevant for the VM phenomenon: CDH5 (also found in our global analysis showed above), *ENG*, *EPHA2*, *KDR* (*VEGFR2*), *LAMC2*, *TEK* and *TIE1* [20,21]. Remarkably, these analyses revealed a significant downregulation of *CDH5*, *KDR* and *TIE1* in MUM-2B ATS1-KO cells (Figure 2c and Figure S1e). We approached a similar study with EL+ C8161 cells, also confirming a significant downregulation of *CDH5* in their respective C8161 ATS1-KO (Figure 2d and Figure S1f). These results indicated that ADAMTS1 exerts a key contribution to EL plasticity in melanoma cells, as its inhibition affected in vitro EL phenotype and downregulated endothelial-related genes, particularly *CDH5*, again highlighting the relevance of this gene.

Next, we approached an in vivo tumor model to shed light on the role of ADAMTS1 in UVM plasticity and tumorigenesis.

### 2.3. ADAMTS1 Inhibition Affects In Vivo Tumor Progression and Vasculature

**Figure 3:**
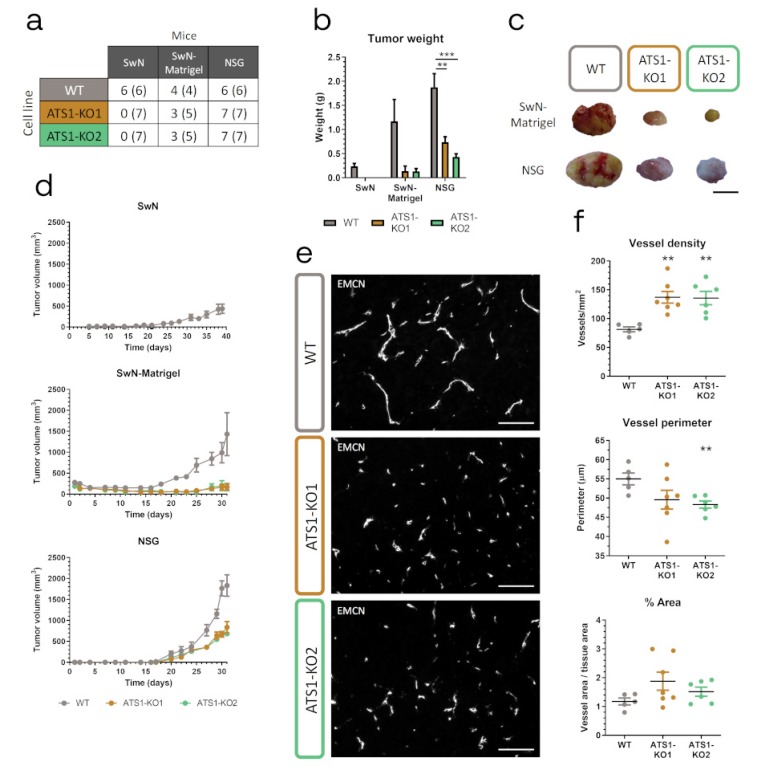
*ADAMTS1* inhibition blocks tumorigenesis and alters tumor vasculature. (**a**) Table indicating the number of mice that developed tumors and the total number of injected mice of every experimental group (in parenthesis); (**b**) Graph representing final tumor weight of different experimental groups, according to panel A; (**c**) Representative pictures of tumors from SwN-Matrigel and NSG groups (black scale bar = 1 cm); (**d**) Graphs representing tumor evolution for each experimental group. (**e**) Representative images of EMCN immunofluorescence analysis of tumor sections from WT and ATS1-KO NSG xenografts (white scale bar = 100 µm); (**f**) Graphs representing tumor vasculature quantification of NSG xenografts: vessel density, vessel perimeter and percentage area (*n* = 5 for WT; *n* = 7 for ATS1-KO1; *n* = 6 for ATS1-KO2). (***, *p* < 0.001 and **, *p* < 0.01. Tumors generated with WT cells were used as control for statistical analyses).

According to previous reports with MUM-2B cells [9], we first executed xenograft studies using Swiss Nude (SwN) mice. Very significantly, all SwN mice injected with WT but none with ATS1-KO cells developed tumors, corroborated even after sacrifice (Figure 3a,b and d). Although this robust result already implied a key contribution of ADAMTS1, we applied the following conditions to confirm it. We injected cells in a 1:1 PBS:Matrigel solution (SwN-Matrigel), reported to support initial cell engraftment and subsequent tumor progression [22]. Under these conditions, ATS1-KO cells, although inducing some tumors, still displayed lower efficiency than WT cells (Figure 3a). Indeed, progression of ATS1-KO-derived tumors was clearly compromised in comparison with WT group (Figure 3b,d).

Given the major blockade of tumor growth when ADAMTS1 was inhibited in these two models, we were unable to get relevant biopsies of some experimental groups. Therefore, we pursued tumor progression on NOD *scid* gamma (NSG) mice, expecting an increased tumor engraftment according to the strongest failure of their immune system [23]. Certainly, now all the injected NSG mice developed tumors (Figure 3a). However, again in accordance with previous results, the comparison of tumor development by WT and ATS1-KO cells in NSG mice revealed significant differences in terms of tumor weight and volume (Figure 3b,d). 

All these data confirmed the relevance of ADAMTS1 for tumor development. Since previous findings already remarked its effect on the vasculature [9,11,24], we analyzed it in our NSG samples. Endomucin (EMCN) IF revealed an increased vessel density but a reduction in perimeter in tumors derived from ATS1-KO cells (Figure 3e,f), resulting in a lack of significant differences in terms of total vessel area between WT and ATS1-KO tumors (Figure 3f), as previously described in other tumor studies [6,9].

At this point, although vasculature displayed alterations, the blockade of tumor progression when ADAMTS1 was inhibited is still unsolved. In line with the recognized plasticity of melanoma cells and the putative role of ADAMTS1 in such phenomena [10], we decided to evaluate stemness-related features in our tumors.

### 2.4. ADAMTS1 Inhibition Compromises Tumor Stemness and Plasticity Features

**Figure 4:**
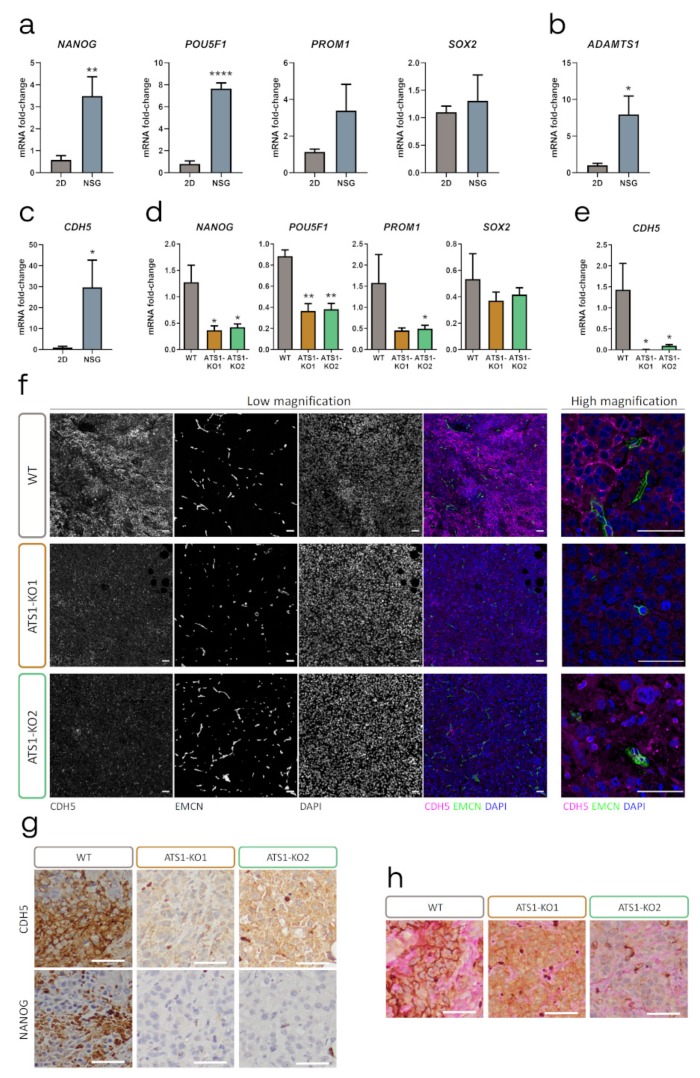
ADAMTS1 inhibition compromises the stemness capacities and endothelial-like phenotype in tumor xenografts. (**a–c**) Graphs representing mRNA fold change expression of *NANOG*, *POU5F1*, *PROM1* and *SOX2* (**a**), ADAMTS1 (**b**) and *CDH5* (**c**), in MUM-2B WT 2D cultured cells and NSG xenografts (*n* = 4–6 for cells and *n* = 3–4 for NSG); (**d-e**) Graphs representing mRNA fold change expression of *NANOG, POU5F1, PROM1* and *SOX2* (**d**), and *CDH5* (**e**) in NSG xenografts generated with WT and ATS1-KO cells (*n* = 3 for WT, *n* = 5 for ATS1-KO1 and *n* = 4–6 for ATS1-KO2); (**f**) Representative images of IF analysis of WT and ATS1-KO NSG xenografts, at low and high magnification. For low magnification, columns from left to right: CDH5, EMCN, DAPI and merge; (**g**) Representative images of IHC staining of CDH5 and NANOG in consecutive sections of WT and ATS1-KO NSG xenografts; (**h**) Representative images of IHC co-staining of CDH5 and PAS, in WT and ATS1-KO NSG xenografts. (****, *p* < 0.0001; **, *p* < 0.01; and *, *p* < 0.05. White scale bar = 50 µm).

Taking advantage of the distinct origin of tumor (human) and stromal (murine) cells in our xenografts, we approached the evaluation of stemness genes *NANOG*, *POU5F1* (*OCT4*), *PROM1* (*CD133*) and *SOX2,* from the tumor origin. Importantly, the analysis of WT tumors revealed a significant upregulation of *NANOG* and *POUF5F1* when compared with the original tumor cells under 2D culture conditions (Figure 4a and Figure S2a), confirming the high impact of the tumor microenvironment on promoting stemness. Interestingly, *ADAMTS1* and *CDH5* also appeared significantly induced in the tumor context (Figure 4b,c, Figure S2b,c), suggesting again their direct link during tumor progression.

In line with the impaired progression, ATS1-KO tumors showed an overall downregulation of stemness markers compared with WT samples, especially significant for the more abundant *NANOG* and *POU5F1* (Figure 4d and Figure S2d). Moreover, in the same way that we observed a significant alteration of endothelial genes in ATS1-KO cultured cells (Figure 2c), now ATS1-KO tumors showed a chief reduction in *CDH5* mRNA levels compared with WT samples (Figure 4e, and Figure S2e). The contribution of CDH5 in the VM phenomenon has already been reported [25,26,27], so we were interested in visualizing human CDH5 in our tumor histological sections by IF. The analysis of WT tumors allowed the identification of a cell surface pattern for CDH5, noticeably associated with tumor cells in the vicinity of EMCN-positive vascular niches (Figure 4f). Furthermore, this pattern was more arduous to find in ATS1-KO tumors, and it was generally less consistent that in WT samples. Therefore, according to the similar behavior of *CDH5* and *NANOG* in our melanoma culture model, we evaluated their relationship in tumor sections. As the co-staining was not feasible, we performed IHC in sequential tumor sections (Figure 4g). In addition to the clearly compromised NANOG positive staining in ATS1-KO tumors, we detected a spatial coincidence of NANOG and CDH5 expression in WT samples that was difficult to appreciate in ATS1-KO sections. Finally, we performed double CDH5-PAS immunostaining to check a similar spatial coincidence between endothelial-related CDH5 and PAS-positive patterns, widely used as identifier of VM [13]. Certainly we observed such concurrence much better in WT than in ATS1-KO tumors, as well as a significant increase in VM+ patterns in WT tumors (Figure 4h).

All these results suggested a close association between stemness and plasticity features that would lead to VM events, so we evaluated such parameters back in tumor cells.

### 2.5. ADAMTS1 Inhibition Compromises In Vitro Stemness Capacities

**Figure 5:**
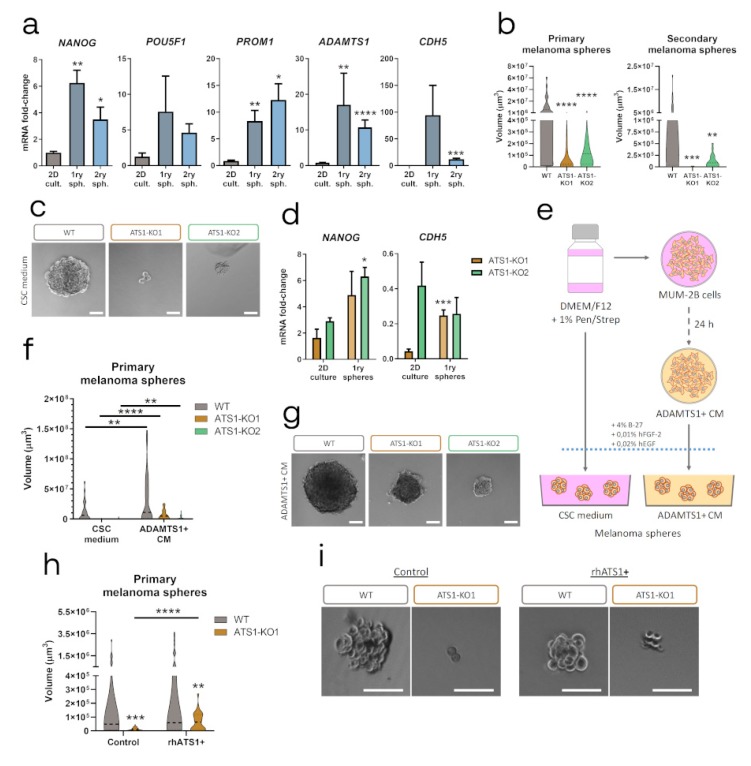
Inhibition of ADAMTS1 compromises melanoma sphere formation. (**a**) Graphs representing mRNA fold-change expression of *NANOG, POU5F1, PROM1*, *ADAMTS1* and *CDH5* in MUM-2B WT 2D culture, primary and secondary melanoma spheres (*n* = 4 for 2D culture, except for *ADAMTS1* which is 12; *n* = 2–5 for primary spheres, and *n* = 4–6 for secondary spheres). 2D cultured cells were used as control for statistical analyses; (**b**) Violin plots representing volume of primary (*n* = 49 for WT, *n* = 62 for ATS1-KO1, and *n* = 58 for ATS1-KO2) and secondary (*n* = 46 for WT, *n* = 34 for ATS1-KO1 and *n* = 21 for ATS1-KO2) melanoma spheres. WT spheres volume was used as control for statistical analyses; (**c**) Representative images of WT and ATS1-KO primary melanoma spheres grown in CSC medium; (**d**) Graphs representing mRNA fold-change expression of *NANOG* and *CDH5* in 2D culture (*n* = 4–6 for ATS1-KO1 and *n* = 2–4 for ATS1-KO2) and primary melanoma spheres (*n* = 2 for ATS1-KO1 and *n* = 4–7 for ATS1-KO2). 2D cultured conditions were used as control for statistical analyses; (**e**) Schematic protocol for melanoma spheres formation, using CSC medium and ADAMTS1+ CM; (**f**) Violin plots representing volume of WT and ATS1-KO primary melanoma spheres grown in CSC medium (*n* = 49 for WT, *n* = 62 for ATS1-KO1, and *n* = 58 for ATS1-KO2) or in ADAMTS1+ CM (*n* = 44 for WT, *n* = 48 for ATS1-KO1, and *n* = 49 for ATS1-KO2); (**g**) Representative images of WT and ATS1-KO primary melanoma spheres in ADAMTS1+ CM; (**h**) Violin plots representing WT and ATS1-KO1 primary melanoma spheres, grown in control CSC (*n* = 27 for WT, and *n* = 44 for ATS1-KO1) or rhATS1+ CSC medium (*n* = 35 for WT, and *n* = 28 for ATS1-KO1); (**i**) Representative images of WT and ATS1-KO1 primary melanoma spheres grown in control and rhATS1+ CSC medium. (****, *p* < 0.0001; ***, *p* < 0.001; **, *p* < 0.01; *, *p* < 0.05. Violin plots indicate the median of every experimental group. White scale bar = 100 µm).

Although stemness features have been attributed to melanoma, our evaluation of stemness genes (*NANOG*, *POU5F1*, *PROM1* and *SOX2*), revealed a very low expression among the tested cells on 2D culture. In agreement with the recognized induction of stemness properties, we approached the melanoma sphere formation assay with MUM-2B cells. Furthermore, we evaluated the expression levels of stemness genes in this material, as well as *ADAMTS1* and *CDH5*, according to their relevance suggested by our previous results. First, we confirmed a significant induction of stemness *NANOG*, *POU5F1* and *PROM1* in the melanoma sphere formation process, in both primary and secondary WT spheres (Figure 5a and Figure S3a). Importantly, we detected a significant induction of both *ADAMTS1* and *CDH5*, implying again a role for these molecules in melanoma plasticity. With these premises, we generated melanoma spheres with ATS1-KO cells. Remarkably, the absence of ADAMTS1 had a dramatic effect by compromising the formation of both primary and secondary spheres (Figure 5b,c and Figure S3b).

Given the strong impairment of ATS1-KO cells to form spheres, we evaluated gene expression levels of *NANOG* and *CDH5* in their primary spheres, according to their alteration in our tumor model. Contrary to WT cells (Figure 5a), ATS1-KO spheres presented no significant changes in the expression of these genes when comparing with 2D cultures (Figure 5d and Figure S3c). It was noteworthy that the low levels of *CDH5* that ATS1-KO cells displayed in 2D culture were not induced at all in the process of sphere formation, as occurred with WT cells. 

All these data demonstrated that ADAMTS1 inhibition also compromises stemness capacities in MUM-2B UVM cells. According to the extracellular nature of ADAMTS1, we then evaluated if medium containing this secreted protease could affect the sphere formation ability. First, we used CM of MUM-2B cells (identified as ADAMTS1+ CM) (scheme in Figure 5e). Notably, this medium enhanced the formation of spheres in both WT and ATS1-KO cells. Indeed, the size and number of spheres of ATS1-KO cells with ADAMTS1+ CM was comparable to that of WT cells with normal CSC medium (Figure 5f,g), suggesting that secreted ADAMTS1 have a positive effect on the recovery of stemness capacities of MUM-2B cells. Second, according to the complex nature of the CM, we performed the sphere assay using CSC medium with and without pure recombinant human ADAMTS1 (rhATS1). Now this experiment revealed that rhATS1 in the medium did not affect WT sphere formation, but it had a significant effect on the sphere formation of ATS1-KO1 cells, although still not reaching WT properties (Figure 5h,i).

While our findings with melanoma spheres confirmed the implication of recognized stemness markers, we were positively surprised that *ADAMTS1* also appeared to be induced during the process and, indeed, that its inhibition blocked the formation of spheres. Furthermore, the induction of endothelial *CDH5* in melanoma spheres revealed an unexpected link between melanoma stemness features and its putative endothelial-like phenotypic properties, supporting the results obtained with our tumor models.

### 2.6. Relevance of Endothelial-like Plasticity in Human Uveal Melanoma

**Figure 6:**
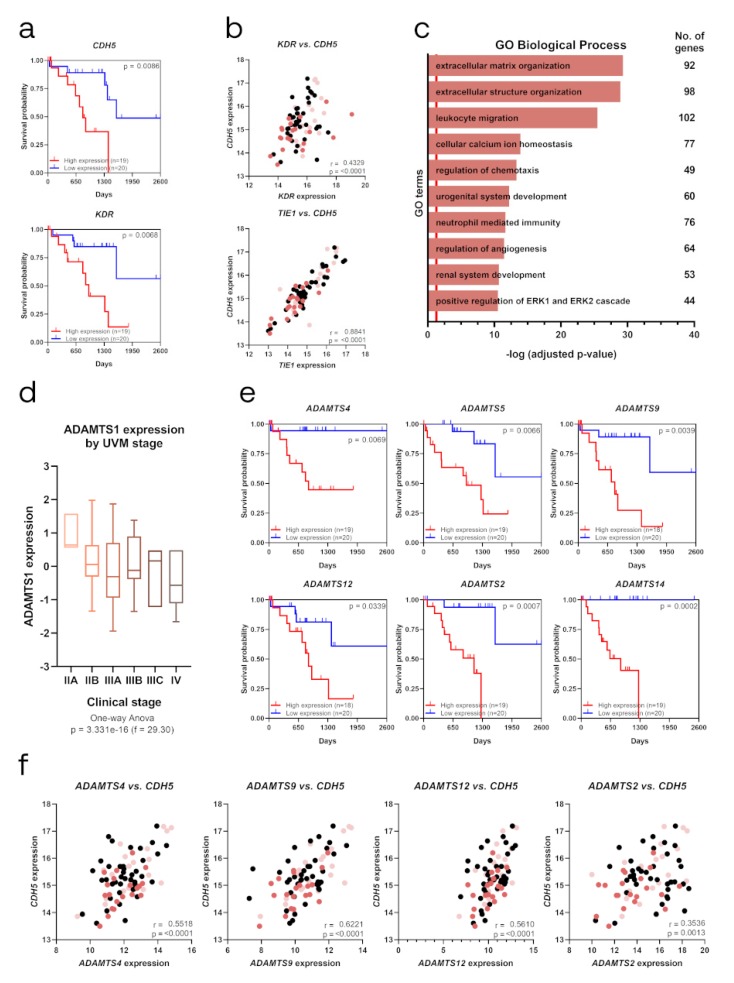
Identification of EL and ECM molecules as poor prognosis factors in TCGA Uveal Melanoma Project (TCGA-UVM). (**a**) Kaplan–Meier survival curves for low and high gene expression levels of EL markers *CDH5* and *KDR*; (**b**) Scatter plot representing Pearson correlation analysis between gene expression levels of *CDH5* and endothelial-related *KDR* and *TIE1*; (**c**) Representation of top ten GO Biological Processes after enrichment analysis using genes that positively correlated (q-value < 0.05) with *CDH5*. Red line determined the limit of significance: -log (0.05); (**d**) Box graph representing *ADAMTS1* expression among different clinical stages of human uveal melanoma, from stage IIA to IV (*n* = 4 for IIA, *n* = 32 for IIB, *n* = 27 for IIIA, *n* = 10 for IIIB, *n* = 3 for IIIC and *n* = 4 for IV); (**e**) Kaplan–Meier survival curves for low and high gene expression levels of extracellular proteases *ADAMTS4, ADAMTS5, ADAMTS9, ADAMTS12, ADAMTS2* and *ADAMTS14*; (**f**) Scatter plot representing Pearson correlation analysis between gene expression levels of *CDH5* and extracellular proteases *ADAMTS4*, *ADAMTS9*, *ADAMTS12* and *ADAMTS2*. (Survival probability is depicted in correlation analysis plots (panels B and F) with light and dark red dots, representing low and high survival probability, respectively. r = Pearson correlation coefficient).

Consistent with the alterations of EL-related and stemness markers described above, all of them modulated by ADAMTS1, we approached a close study of their contribution in human UVM. Considering the infrequent nature of this tumor, the public availability of gene expression data in the TCGA-UVM Project [28] allowed us to pursue such analyses in a relevant number of samples, including prognosis parameters and staging classification. First, using the UCSC Xena platform, we found that endothelial-related *CDH5* and *KDR* appeared as significant poor prognosis factors (Figure 6a), in full agreement with our experimental observations. Furthermore, the evaluation of co-expressing genes across these cohorts of patients revealed a higher and significant correlation of *CDH5* with additional endothelial-related genes like *CD34*, *TIE1*, *FLT4*, *KDR* and *COL4A1* (Figure 6b and Figure S4), confirming the strong association of endothelial-related signature in this neoplasia. According to the chief role of CDH5, we then approached a GO-enrichment analysis using the list of significantly positive correlated genes with *CDH5* (Table S4a, 1196 genes). This exploration revealed key features such as ECM organization and the regulation of angiogenesis (Figure 6c and Table S4b), highlighting the close and significant relationship between endothelium and matrix remodeling.

Then, taking into consideration the modulatory role that extracellular proteases exert on tumor plasticity, we approached a whole evaluation of all members of the ADAMTS family of proteases, noticing the strong similarity that exists among them at the structural and functional levels. In agreement with the study of stemness markers, we did not find *ADAMTS1* correlating with survival in UVM patients, which was probably related with their role in initiating stages. Indeed, when we analyzed *ADAMTS1* expression data considering different stages of tumor progression, we observed a clear tendency for *ADAMTS1* to be more expressed at early phases (identified here with stage IIA) and decreases with higher grades (Figure 6d). This higher expression at initial stages of UVM would support our experimental data, as we observed a link between ADAMTS1 and stemness features, closely related with tumor initiation and early UVM progression. Furthermore, it resulted quite appealing the fact that up to six related family members displayed significant values as poor prognosis factors for UVM, including the closer *ADAMTS4*, *ADAMTS5* and *ADAMTS9*, but also others like *ADAMTS12, ADAMTS2* or *ADAMTS14* (Figure 6e). Going back to ADAMTSs actions and *CDH5* induction, we observed positive significant correlations between some of these proteases and *CDH5* (Figure 6f), supporting the robust association between endothelial-like plasticity and ADAMTSs activity favoring UVM progression.

## 3. Discussion

Tumor microenvironment (TME) remodeling is being recognized as a relevant contributor to complex tumor heterogeneity, and one to be researched for a better understanding. To date, although a number of extracellular proteases have been identified as possible therapeutic targets due to their actions during tumor growth, their clinical use still requires much deeper investigation. In this work, we first focused our attention on the impact of the extracellular protease ADAMTS1 on human uveal melanoma (UVM), and particularly on its effects on cancer plasticity phenomena, revealing a new relationship between intrinsic endothelial-like (EL) properties on tumor cells and their enrichment on stemness features. A combination of experimental in vitro and in vivo approaches, together with the use of bioinformatics methodologies on public tumor databases, allowed us to unveil the unexplored relevance of endothelial-specific molecules, such as CDH5, strongly correlating with ADAMTS proteases.

Our initial and comprehensive characterization of human uveal and skin melanoma cell lines confirmed their heterogeneity but, more importantly, it supported their classification as EL+ or EL−. Although based on experimental phenotypic properties, our bioinformatics approach using available datasets of melanoma cell lines showed a clear connection with vascular functions as coagulation and hemostasis. This approach also highlighted the contribution of endothelial *CDH5* as a key gene supporting our own data. While the literature has already shown the existence of differential gene signatures among melanoma cell lines with different aggressiveness [15,29], our methodology discriminated according to their behavior in the recognized Matrigel assay. In addition, we detected *ADAMTS1* to be significantly upregulated in EL+ cells. The absence of *ADAMTS1* in our in silico results, as also occurred with stemness related molecules, is probably linked with their low expression levels, making them undetectable for microarray procedures.

Focusing on UVM, we succeeded in inhibiting ADAMTS1 in MUM-2B cells, and our subsequent studies confirmed the disruption of relevant tumorigenic features. First, our Matrigel assays to evaluate the EL phenotype showed that ATS1-KO cells were compromised in comparison with WT cells. Moreover, we detected in these deficient cells a clear downregulation of EL markers, already associated with VM and its aggressive phenotype in melanoma [15,27,30]. Notably, the performance of xenograft assays in distinct mouse models supported the pro-tumorigenic effects of ADAMTS1 in UVM, revealed mainly by the significant blockade of tumor development when ADAMTS1 was inhibited. Indeed, our data with SwN mice disclosed a complete halt using ATS1-KO cells, which was partially overcome using an NSG model. Importantly, the immune system of NSG mice displays a stronger defect than SwN, so the distinct ability of ATS1-KO cells to initiate tumor progression in these two models could be related with an immunomodulatory role already attributed to ADAMTS1 [11]. Certainly, these results encourage further studies to unveil the impact of this ECM-modifying enzyme on the immune system, although distinct models must be considered.

Regarding the tumor vasculature, in agreement with previous reports [6,9], this current study also showed evocative alterations although the overall vasculature appeared normalized. These observations are aligned with the widely described role of some ADAMTSs as modulators of vasculature [24]. Nonetheless, additional actions of ADAMTS1 contribute to tumor development, at least in this setting. Very importantly, we were able to match alterations of endothelial and stemness parameters. To our knowledge, this is the first time where a tumor displayed a clear correlation between the acquisition of endothelial-like features and an intrinsic stemness signature, indeed correlating with the disruption of tumorigenic properties. Our studies revealed an overall increase in stemness genes in tumors in comparison to the original 2D cultures. Furthermore, we also found the EL marker *CDH5* to be similarly regulated. This intimate relationship between stemness, plasticity and the acquisition of EL properties, both in vitro and in vivo, with everything modulated by the protease ADAMTS1, emphasizes the chief contribution of ECM modifying enzymes. Suggestively, these results encourage the design of new and improved specific inhibitors of matrix metalloproteases to overcome former disappointing results in clinical trials [31]. Indeed, our results support a main involvement of CDH5-related pathways, but not strictly from endothelial origins as originally hypothesized, according to their relevant expression in plastic tumor cell populations.

Our assays involving the formation of tumor spheres also reinforced our findings. Although the development of melanoma spheres from the MUM-2B cell line was already reported [32], to our knowledge, this is the first work including a deeper description and characterization of the resulting spheres. WT cells showed a clear upregulation of main stemness markers, together with the newly reported induction of *CDH5* and the protease *ADAMTS1*. While CDH5 has been already related with stemness parameters [17,33,34] as a causal factor of vasculogenic mimicry events, our data exposed that extracellular proteases such as ADAMTS1 should also been considered when studying the stemness capacities or the aggressiveness of a tumor. In the case of ATS1-KO spheres, not only were they unable to generate optimal spheres, but also, there were no significant changes to the expression of our genes of interest. Importantly, our experiments using ADAMTS1-enriched media (secreted in the CM of melanoma cells or as exogenous recombinant protein) supported the extracellular contribution of this protease as a key factor for tumor initiation and growth, indeed corroborated by our in vivo approaches. This role of ADAMTS1 during initiating stages of tumorigenesis possibly justifies its absence in standard gene expression notations as we mentioned in our studies in cell lines and also in human UVM samples.

According to the modest knowledge of UVM, considered a rare type of cancer with an estimated incidence of 4.9–5.2 cases per million in the United States and a rate of 2–8 cases per million in Europe [17], our new findings give strength to its recognized plastic nature. With the intention to advance the fight against this type of melanoma, we conducted a deep study of genes of interest in human UVM datasets from TCGA. Suggestively, we found that the endothelial-related genes *CDH5* and *KDR* appeared as significant poor prognosis factors in these neoplasias. Although the fact that *CDH5* should be considered a poor prognosis gene is supported all along this work, we find it especially interesting that other EL genes displayed similar features. Indeed, these genes were formerly related with VM and stemness features [30,35], and our own experiments confirm such involvement.

Very importantly, the evaluation of *ADAMTS1* in this tumor collection allowed us to observe its higher expression levels at early phases of this malignant tumor, and then appeared downregulated as the disease progressed to advanced stages, in harmony with our results on stemness features. Indeed, it resulted quite appealing the fact that such positive association did occur with high expression of other ADAMTS proteases (*ADAMTS2*, *ADAMTS4*, *ADAMTS5*, *ADAMTS9*, *ADAMTS12* and *ADAMTS14*) that definitively implied a key role of these proteases during melanoma progression. We need to remark on the strong similarity among these members, perfectly complementary within the highly complex scenario of the TME.

Our in vitro and in vivo findings expose a complex communication between the endothelial phenotype, stemness features and ECM regulation, contributing to the final fate of the tumor. More specifically, the requirement of *ADAMTS*1 for tumor progression in our mouse models, its relationship with stemness and endothelial plasticity features, and its higher expression at earlier stages of UVM, all support the role of *ADAMTS1* as a pro-tumorigenic factor for this rare type of tumor. Lastly, this association suggests the participation of key molecules at the cell-matrix and cell-cell boundary connecting the proteolytic action of ADAMTS proteases with transmembrane and intracellular pathways, which full comprehension would help to define new therapeutic strategies.

## 4. Materials and Methods

### 4.1. Cell Culture and Generation of ADAMTS1-Knockout Cells

Uveal melanoma MUM-2B and MUM-2C, and skin melanoma C8161 cell lines were kindly provided by Dr. Arjan W. Griffioen (VUmc, Amsterdam, The Netherlands); skin melanoma SK-MEL-28, SK-MEL-103, SK-MEL-147 by Dr. Juan A. Recio (VHIR, Barcelona, Spain); A-375 and G-361 by Dr. Javier Oliver (IPBLN-CSIC, Granada, Spain); and HEK293T by Dr. Pablo Menéndez (IJC, Barcelona, Spain). All cell lines were cultured in the appropriate medium supplemented with 10% fetal bovine serum (Gibco) and 1% Penicillin/Streptomycin solution (Biowest, Nuaillé, France), under standard conditions (37 °C, 5% CO_2_ and 95% relative humidity). Specific media were: RPMI 1640 with stable glutamine (Biowest, Nuaillé, France) for MUM-2B, MUM-2C and C8161; High Glucose Dulbecco’s Modified Eagle Medium (DMEM) with stable glutamine and sodium pyruvate (Biowest, Nuaillé, France) for A-375, G-361, SK-MEL-28, SK-MEL-103, SK-MEL-147 and HEK293T. All cell lines were routinely tested for *Mycoplasma* (Venor^®^GeM qEP, Minerva Biolabs, Berlin, Germany). MUM-2B cell line was authenticated by STR Profiling (AmpFLSTR^®^ Identifiler^®^ Plus, Applied Biosystems, Waltham, MA, USA).

The generation of ATS1-KO cells with lentivirus-based CRISPR-Cas9 system has been described [36]. Two clonal populations were obtained from MUM-2B cell line (named ATS1-KO1 and ATS1-KO2) and other two from C8161 (named ATS1-KO3 and ATS1-KO4), after two weeks of selection (0.25 µg/mL Puromycin), and single cell cloning isolation and expansion processes. They were subjected to Sanger DNA sequencing (primer sequences in Table S1) and Western blot analysis to confirm gene edition and inhibition, respectively.

### 4.2. In Vitro 3D Matrigel-Based Assay

35 µL/well of Matrigel (Corning, Corning, NY, USA) were dispensed in a 96-well plate kept on ice to avoid gelling. After Matrigel gelling, 100 µL of serum-free medium were added to each well. Finally, 100 µL of serum-free medium containing cells (20,000–30,000 cells) were added. Follow-up was performed taking pictures at various time points (Axio Vert microscope, A-Plan 5x/0.12 objective, Zeiss, Oberkochen, Germany). If appropriate, 24 h pictures were subjected to WimTube analysis (Wimasis, Córdoba, Spain) as indicated [37].

### 4.3. Tumor Xenograft Assays

Female Swiss Nude (SwN) and NOD *scid* gamma (NSG) mice were purchased from Charles River Laboratories and housed at CIBM-UGR animal facility according to institutional guidelines (Approved Ethical Committee #152-CEEA-OH-2016). For xenograft generation, 1 × 10^6^ cells in 100 µL PBS were subcutaneously injected in the flank of 6-weeks old mice. When using Matrigel as scaffold, 2 × 10^6^ cells in a 150 µL PBS:Matrigel dilution (1:1) were subcutaneously injected in the flank of 16-weeks old SwN mice. Animals were monitored every two days after cell injection until final time point, when they were sacrificed and tumors were dissected for further analyses. Tumor volume was calculated as: *in progress tumor volume = (π × length × width^2^)/6*, and *final tumor volume = (π × length × width × height)/6*) [11].

### 4.4. Vasculature Characterization and Immunohistochemistry

For the morphometric analysis of vasculature, tumor paraffin sections were subjected to immunofluorescence (IF) with a rat anti-mouse EMCN (endomucin) antibody (V.7C7, SC-65495, SCBT) and Alexa Fluor 488 donkey anti-rat secondary antibody (A21208, ThermoFisher Scientific, Waltham, MA, USA). IF images were captured (Axio Imager A.1 microscope, EC Plan-Neofluar 10x/0.30 Ph 1 objective, AxioCam MR R3, Zeiss, Oberkochen, Germany) and converted to binary for further analysis as indicated [9]. CDH5 and NANOG were detected with rabbit anti-human CDH5 (160840, Cayman Chemical, Ann Arbor, MI, USA) or NANOG (4903, Cell Signaling Technology, Danvers, MA, USA) antibodies, respectively. Alexa Fluor 647 goat anti-rabbit (A-21245, ThermoFisher Scientific, Waltham, MA, USA), and Dako Envision^TM^ + System-HRP (DAB) (K4010, Agilent Technologies, Santa Clara, CA, USA) were used as secondary antibodies. Antigen retrieval was performed by slide immersion in Tris-HCl 0.5 M pH = 10 solution and 10 min boiling. DAPI (D8417, Sigma-Aldrich, St. Louis, MO, USA) solution and Dako Mayer’s Hematoxylin (S3309, Agilent Technologies, Santa Clara, CA, USA) were used for nuclear counterstain in IF and IHC, respectively. Antifade Mowiol (81381, Sigma-Aldrich, St. Louis, MO, USA)-DABCO (D27802, Sigma-Aldrich, St. Louis, MO, USA) mixture and D.P.X. (317616, Sigma-Aldrich, St. Louis, MO, USA) were used as mounting media for IF and IHC, respectively. PAS staining was performed with Periodic acid solution (3951, Sigma-Aldrich, St. Louis, MO, USA) and Schiff’s reagent (3952016, Sigma-Aldrich, St. Louis, MO, USA). Confocal images were captured with a LSM 710 Axio Observer (Plan-Apochromat 63x/1.4 Oil DIC M27 objective, Zeiss, Oberkochen, Germany). IHC images were captured with a BX43 microscope (Plan-Achromat 20x/0.4 objective, Olympus, Tokyo, Japan).

### 4.5. Melanoma Sphere Formation Assay

5000 cells/mL were seeded in non-adherent bacterial plates with CSC medium (DMEM-F12 without L-Glutamine nor Hepes (Biowest, Nuaillé, France) supplemented with 1% Penicillin/Streptomycin (Biowest, Nuaillé, France), B-27™ (40 mL/L, ThermoFisher Scientific, Waltham, MA, USA), human FGF-2 (0.01 µg/mL, Miltenyi Biotec, Bergisch Gladbach, Germany) and human EGF (0.02 µg/mL, Miltenyi Biotec, Bergisch Gladbach, Germany). Medium was renewed weekly by low speed centrifugation (5 min, 800 rpm) until primary spheres were obtained after 3 weeks. To generate secondary spheres, primary spheres were disaggregated through a 29G needle and seeded again at a density of 5000 cells/mL in 60 mm suspension culture dishes (Corning, Corning, NY, USA). For assays using recombinant human ADAMTS1 (rhATS1, 2197-AD, R&D), spheres were grown in 6-well ultralow attachment plates (Corning, Corning, NY, USA), using CSC medium supplemented with 1 µg/mL rhATS1. For assays using conditioned medium (CM), fresh medium was collected from 24 h cultured MUM-2B cells and supplemented with B-27, FGF-2 and EGF as described above. Images were captured with an Axio Vert microscope (A-Plan 5x/0.12 objective, Zeiss, Oberkochen, Germany), and evaluated with Carl Zeiss ZEN 2.3 SP1 (black) software (Oberkochen, Germany). Sphere volume was calculated as: *sphere volume = (π × length × width^2^)/6*.

### 4.6. RNA Isolation and Quantitative RT-PCR

RNA was extracted with NucleoSpin® RNA kit (Macherey-Nagel, Duren, Germany) and reverse transcribed with iScript™ cDNA Synthesis Kit (Bio-Rad, Hercules, CA, USA). qPCR reactions were performed with Fast SYBR™ Green Master Mix (Applied Biosystems, Waltham, MA, USA), using 7900HT Fast Real-Time PCR (Applied Biosystems, Waltham, MA, USA) and QuantStudio 6 Flex Real-Time PCR (Applied Biosystems, Waltham, MA, USA) platforms. *ACTB*, *B2M* and *RNA18S1* were used as housekeeping genes, depending on the sample origin and the platform (primer sequences in Table S1).

### 4.7. Western Blot Analysis

Secreted proteins were obtained from CM of melanoma cells cultured over 24 h in the absence of serum, and concentrated with StrataClean resin (Agilent Technologies, Santa Clara, CA, USA) [38]. Total protein from cell lysates was extracted with RIPA buffer (containing 10 µg/mL aprotinin, 1 mM PMSF and 100 µM leupeptin) and quantified with Pierce BCA Protein Assay Kit (ThermoFisher, Waltham, MA, USA) in an Infinite 200 PRO NanoQuant (Tecan, Männedorf, Switzerland) absorbance reader. All samples (CM from cells cultured in a 100 mm dish, and 30 µg of cell lysate) were resolved by SDS-PAGE and transferred to PVDF membranes (Bio-Rad, Hercules, CA, USA). Membranes were stained with a Red Ponceau solution to visualize loaded proteins. Then, membranes were blocked with 5% low-fat milk and incubated with sheep anti-human ADAMTS1 (AF5867, R&D Systems, Minneapolis, MN, USA) and monoclonal mouse anti-Actin (sc-8432, Santa Cruz Biotechnology, Dallas, TX, USA) antibodies. After incubation with HRP-conjugated secondary antibodies (HAF016, R&D Systems, Minneapolis, MN, USA), signal was detected with the ECL Prime Western Blotting Detection Reagent (GE Healthcare Life Sciences, Marlborough, MA, USA) in an ImageQuant LAS4000 (GE Healthcare Life Sciences, Marlborough, MA, USA).

### 4.8. Bioinformatic Analyses of Cell Lines Datasets and Melanoma Patients

NCBI Gene Expression Omnibus [39] was mined to find optimal datasets of melanoma cell lines used in this study plus human umbilical vein endothelial cells (HUVECs), yielding a total of 31 samples from 13 different datasets (Table S2). Gene expression data were downloaded, probe identifiers were annotated with gene symbols and median gene expression values were calculated for duplicated genes and rank normalized to allow sample comparisons from different technologies. Linear models implemented in limma R package [40] allowed us to analyze differential gene expression between groups of cell lines. Genes were selected by false discovery rate (FDR) < 0.05. A combination of clusterProfiler [41] and REViGO [42] were used to evaluate representative enriched GO biological processes, selecting GO terms showing an adjusted *p*-value < 0.05.

Genomic Data Commons TCGA Uveal Melanoma Project (TCGA-UVM) [28,43] and UCSC Xena platform [44] were used to obtain survival and gene expression data from patients. Kaplan–Meier curves were obtained comparing patients with low (quartile 1 [Q1]) and high (above quartile 3 [Q3]) gene expression levels. Pearson correlation analyses were performed using the list of genes that positively correlate with *CDH5* using cBioPortal platform [45,46], and comparing normalized gene expression levels from Xena platform. Gene expression data were shown as Fragments Per Kilobase of transcript per Million mapped reads upper quartile (FPKM-UQ).

### 4.9. Statistical Analysis

Statistical analyses were made with GraphPad Prism 8 (GraphPad software Inc., San Diego, CA, USA). Except when indicated, graphs represent *mean* ± *SEM*, and unpaired t tests were performed to compare means of two experimental groups. Robust regression and Outlier removal (ROUT) method was applied to identify outliers when necessary. For qPCR analysis, values of ∆∆Ct out of *mean* ± *SD* were considered as outliers, as well as values of Ct that were lower than *Q1* − *1.5* × *IQR* (interquartile range) or greater than *Q3* + *1.5* × *IQR*.

## 5. Conclusions

In this work, we find that the inhibition of the extracellular protease ADAMTS1 alters the endothelial-like properties of uveal melanoma cells in vitro and affects tumor vasculature in vivo. Indeed, our experiments show that ADAMTS1 is also relevant to the stemness capacities of such tumor cells in both in vitro and in vitro approaches, with strong consequences for tumor growth. The inhibition of this protease reveals a new link between stemness and endothelial-like features of uveal melanoma cells, and the evaluation of gene expression data of human samples shows its relevance at early stages, also implying the contribution of further ADAMTS members during more advance stages.

## Supplementary Materials

The following are available online at https://www.mdpi.com/2072-6694/12/4/801/s1, Figure S1: ADAMTS1 expression in human melanoma cell lines and effect of its edition on in vitro EL phenotypic properties; Figure S2: ADAMTS1 inhibition compromises the expression of stemness and EL related genes in tumor xenografts; Figure S3: ADAMTS1 inhibition compromises formation of secondary melanoma spheres; Figure S4: Positive correlation of additional endothelial genes with CDH5; Table S1: Sequences of used primers for genes of interest; Table S2: Public gene expression datasets; Table S3A-B: Differently-expressed genes between EL+ and EL− cells and GO enrichment analysis of upregulated genes in EL+ cells; Table S4A-B: Genes positively correlated with CDH5 and GO enrichment analysis.
