# Supplementary material for: Extracellular Protease ADAMTS1 Is Required at Early Stages of Human Uveal Melanoma Development by Inducing Stemness and Endothelial-Like Features on Tumor Cells
Source: Cancers (Basel). 2020 Mar 27;12(4):801. doi: 10.3390/cancers12040801 (PMC7226337; doi:10.3390/cancers12040801)
Supplement: Supplementary file 1 [file cancers-12-00801-s001.zip › cancers-725454-supplementary/Supplementary File 1 - Supplementary Figure S1.pdf]

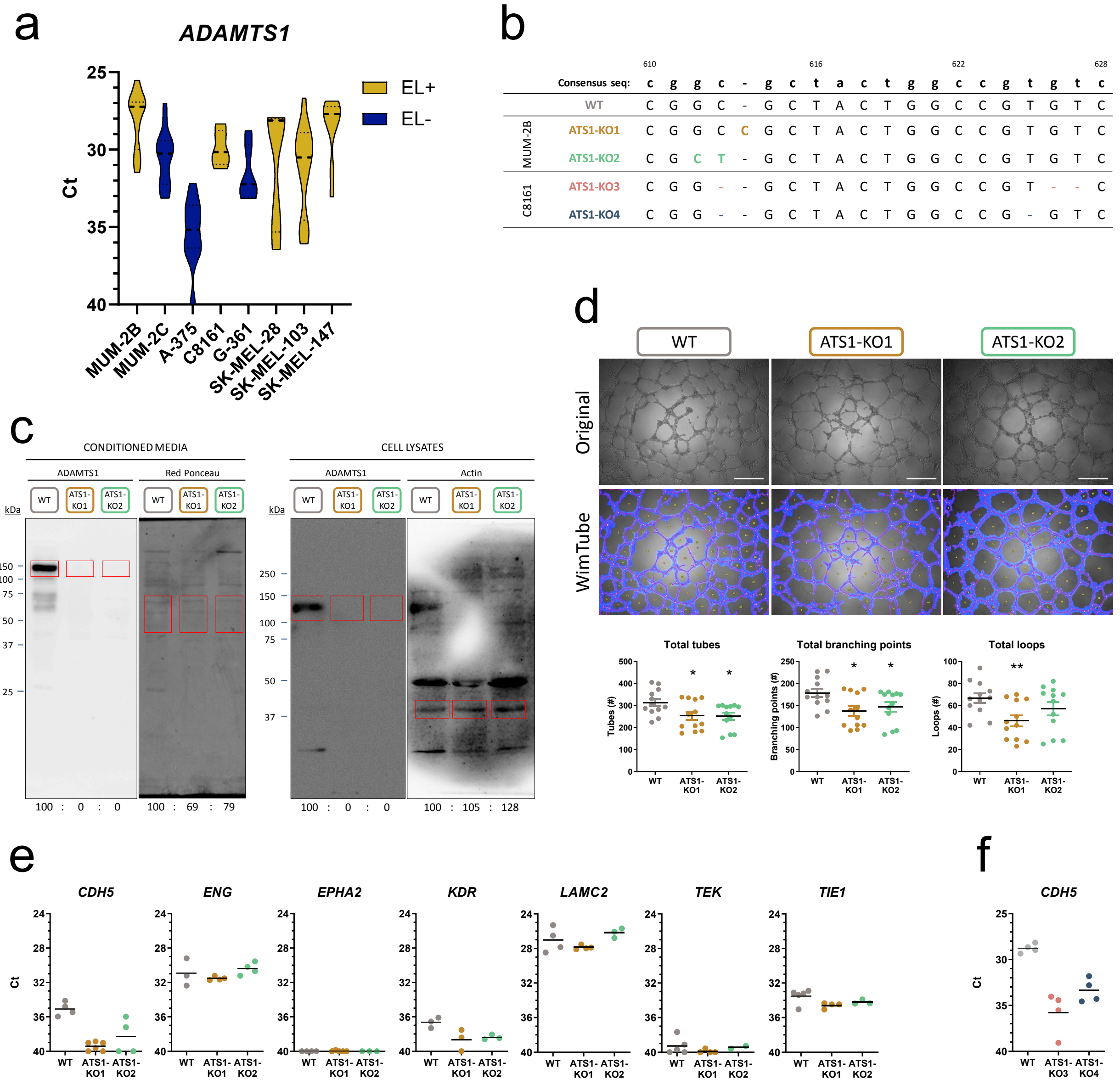

Supplementary Figure S1. ***ADAMTS1*** **expression in human melanoma cell lines and effect of its edition on** ***in vitro*** **EL phenotypic properties.**

(**a**) Graph representing Ct values of *ADAMTS1* in human melanoma cell lines, indicating their EL- or EL+ phenotype (n = 21 for MUM-2B, n = 17 for MUM-2C, n = 11 for A-375, n = 5 for C8161 and SK-MEL-28, n = 6 for G-361, n = 15 for SK-MEL-103 and n = 9 for SK-MEL-147); (**b**) Schematic view of the *ADAMTS1* (NM_006988) gene edition in MUM-2B and C8161 cell lines to obtain ATS1-KO cells. Editions in MUM-2B were: ATS1-KO1, an insertion of a cytosine (C) in position 614; ATS1-KO2, a substitution of a guanine-cytosine (GC) for a cytosine-thymine (CT) in positions 612-613. Editions in C8161 were: ATS1-KO3, deletions in positions 613, 626 and 627; ATS1-KO4, deletions in positions 613 and 625; (**c**) Uncut Western blots from Figure 2a, including the ratio between signals of different lanes at the bottom of the image. Densitometry analysis of highlighted areas was performed using the "Gel, Label Peaks" tool from ImageJ software; (**d**) Representative images (original and WimTube filtered) of Matrigel assay for MUM-2B WT and ATS1-KO cells, 24 h after seeding 30.000 cells/well. Scatter plots represent the parameters: total tubes, total branching points and total loops (n = 12 for all groups, white scale bars = 500 µm); (**e**) Graph representing Ct values of *CDH5*, *ENG*, *EPHA2*, *KDR*, *LAMC2*, *TEK* and *TIE1* in MUM-2B WT and ATS1-KO cells; (**f**) Graph representing Ct values of *CDH5* in C8161 WT and ATS1-KO cells.

**, p < 0,01 and *, p < 0,05. WT cells were used as control for statistical analyses.
