# Supplementary material for: Extracellular Protease ADAMTS1 Is Required at Early Stages of Human Uveal Melanoma Development by Inducing Stemness and Endothelial-Like Features on Tumor Cells
Source: Cancers (Basel). 2020 Mar 27;12(4):801. doi: 10.3390/cancers12040801 (PMC7226337; doi:10.3390/cancers12040801)
Supplement: Supplementary file 1 [file cancers-12-00801-s001.zip › cancers-725454-supplementary/Supplementary File 2 - Supplementary Figure S2.pdf]

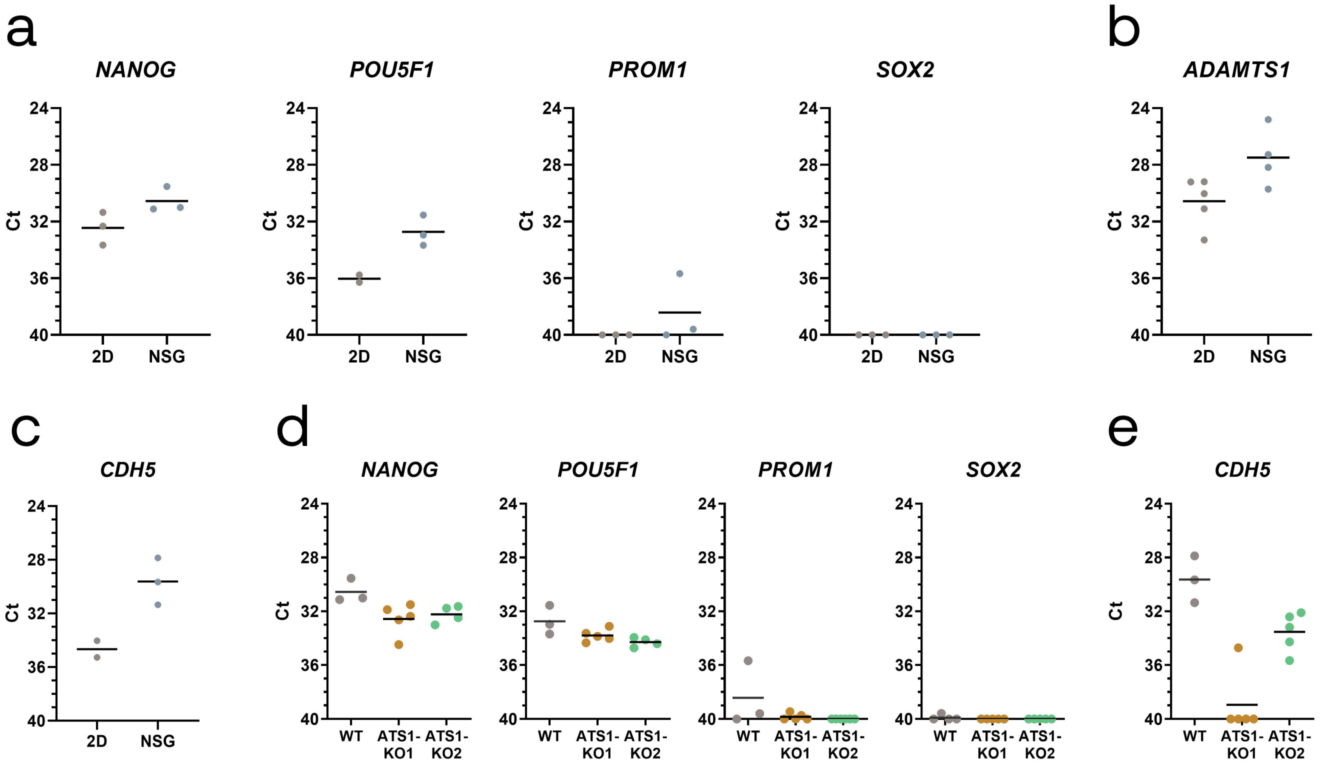

Supplementary Figure S2. ADAMTS1 inhibition compromises the expression of stemness and EL related genes in tumor xenografts.

(**a-c**) Graphs representing Ct values of *NANOG, POU5F1, PROM1* and *SOX2* (**a**), *ADAMTS1* (**b**) and *CDH5* (**c**) in 2D WT cultured cells and their NSG xenografts; (**d-e**) Graphs representing Ct values of *NANOG, POU5F1, PROM1* and *SOX2* (**d**) and *CDH5* (**e**) in NSG xenografts generated with WT and ATS1-KO cells.
