# Supplementary material for: Extracellular Protease ADAMTS1 Is Required at Early Stages of Human Uveal Melanoma Development by Inducing Stemness and Endothelial-Like Features on Tumor Cells
Source: Cancers (Basel). 2020 Mar 27;12(4):801. doi: 10.3390/cancers12040801 (PMC7226337; doi:10.3390/cancers12040801)
Supplement: Supplementary file 1 [file cancers-12-00801-s001.zip › cancers-725454-supplementary/Supplementary File 3 - Supplementary Figure S3.pdf]

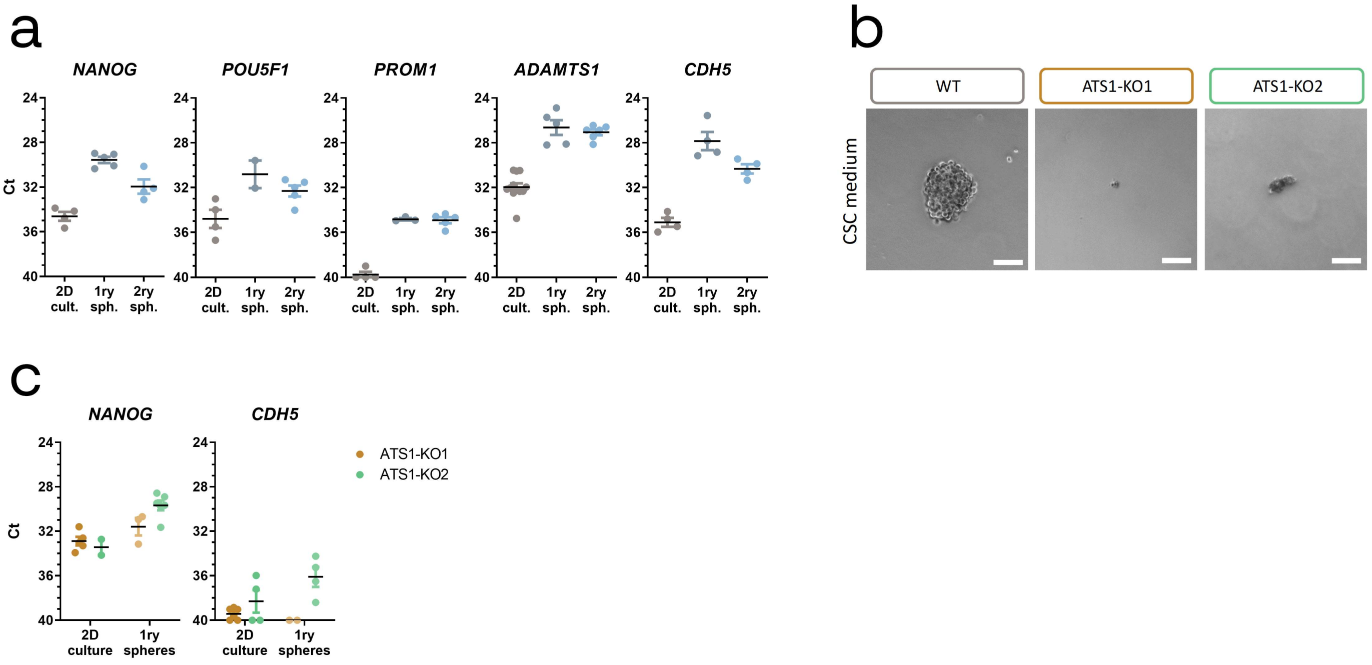

Supplementary Figure S3. ADAMTS1 inhibition compromises formation of secondary melanoma spheres.

(**a**) Graphs representing Ct values of *NANOG*, *POU5F1*, *PROM1*, *ADAMTS1* and *CDH5* in 2D WT cultured cells, primary and secondary melanoma spheres; (**b**) Representative images of secondary melanoma spheres generated from WT, ATS1-KO1 and ATS1-KO2 cells in CSC medium. White scale bar = 100 µm; (**c**) Graphs representing Ct values of *NANOG* and *CDH5* in 2D culture and primary spheres of ATS1-KO cells.
