# Supplementary material for: Extracellular Protease ADAMTS1 Is Required at Early Stages of Human Uveal Melanoma Development by Inducing Stemness and Endothelial-Like Features on Tumor Cells
Source: Cancers (Basel). 2020 Mar 27;12(4):801. doi: 10.3390/cancers12040801 (PMC7226337; doi:10.3390/cancers12040801)
Supplement: Supplementary file 1 [file cancers-12-00801-s001.zip › cancers-725454-supplementary/Supplementary File 4 - Supplementary Figure S4.pdf]

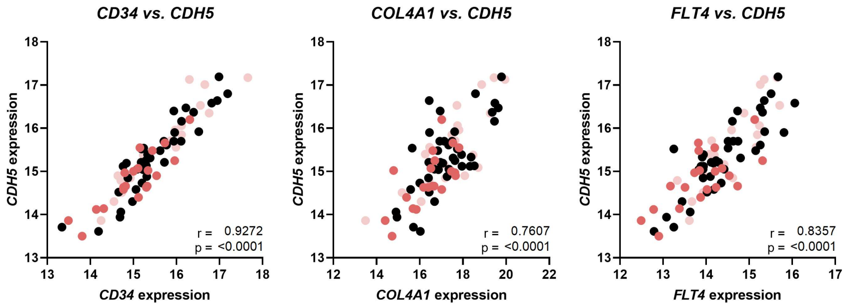

Supplementary Figure S4. Positive correlation of additional endothelial genes whith *CDH5*.

Scatter plots representing Pearson correlation analysis between gene expression levels of *CDH5* and endothelial-related *CD34*, *COL4A1* and *FLT4*. Survival probability is depicted with light and dark red dots, representing low and high survival probability, respectively.
